# A Review of Medicinal Plants of the Himalayas with Anti-Proliferative Activity for the Treatment of Various Cancers

**DOI:** 10.3390/cancers14163898

**Published:** 2022-08-12

**Authors:** Hailemeleak Regassa, Anuradha Sourirajan, Vikas Kumar, Sadanand Pandey, Deepak Kumar, Kamal Dev

**Affiliations:** 1 Faculty of Applied Sciences & Biotechnology, Shoolini University of Biotechnology & Management Sciences, Solan 173229, India; 2 Institute of Biotechnology, Chandigarh University, Mohali 140413, India; 3 Department of Chemistry, College of Natural Sciences, Yeungnam University, 280 Daehak-Ro, Gyeongsan 38541, Korea; 4 Department of Pharmaceutical Chemistry, School of Pharmaceutical Sciences, Shoolini University, Solan 173229, India

**Keywords:** cancer, medicinal plants, phytocompounds, cytotoxicity, immunotherapies, Himalayas

## Abstract

**Simple Summary:**

Drugs are used to treat cancer. Most drugs available in the market are chemosynthetic drugs and have side effects on the patient during and after the treatment, in addition to cancer itself. For instance, hair loss, loss of skin color and texture, loss of energy, nausea, infertility, etc. To overcome these side effects, naturally obtained drugs from medicinal plants are preferred. Our review paper aims to encourage the study of anticancer medicinal plants by giving detailed information on thirty-three medicinal plants and parts that constitute the phytochemicals responsible for the treatment of cancer. The development of plant-based drugs could be a game changer in treating cancer as well as boosting the immune system.

**Abstract:**

Cancer is a serious and significantly progressive disease. Next to cardiovascular disease, cancer has become the most common cause of mortality in the entire world. Several factors, such as environmental factors, habitual activities, genetic factors, etc., are responsible for cancer. Many cancer patients seek alternative and/or complementary treatments because of the high death rate linked with cancer and the adverse side effects of chemotherapy and radiation therapy. Traditional medicine has a long history that begins with the hunt for botanicals to heal various diseases, including cancer. In the traditional medicinal system, several plants used to treat diseases have many bioactive compounds with curative capability, thereby also helping in disease prevention. Plants also significantly contributed to the modern pharmaceutical industry throughout the world. In the present review, we have listed 33 medicinal plants with active and significant anticancer activity, as well as their anticancer compounds. This article will provide a basic set of information for researchers interested in developing a safe and nontoxic active medicinal plant-based treatment for cancer. The research will give a scientific foundation for the traditional usage of these medicinal herbs to treat cancer.

## 1. Introduction

Cancer is different from microbe-related diseases caused by parasites, and environmental and infectious diseases caused by antigens foreign to our body system. There are various factors for the development of cancers in humans when epigenetics or genetic factors lead to the mutation of the normal cells [1]. Epigenetics is the study of changes in heritable gene expression that lead to the proliferation of abnormal cells [2]. Cancer is caused by the abnormal function of the genes and the manipulated pattern of gene expression, loss of the process of normal cell growth, development, and control; malfunctioning of apoptosis; initiation of angiogenesis, and metastasizing to other healthy tissue or organs [3]. The spread of cancer from its cells or tissue of origin to another healthy part of tissues or organs is called metastasis [4]. According to the ancient Greeks, cancer was considered as constitutional melancholy and black bile. Various cancer research bodies give first attention to early diagnosis, prevention, and treatment of cancer [5]. According to GLOBOCAN esti-ma-tes, there will be 28.4 million new cases of cancer worldwide in 2040, a 47 percent increase over the average number of cases in the year 2020. A significant increase in the rate of transitioning is anticipated due to demographic shifts, from 65 to 95 percent vs. 32 to 56 percent transitioned, and this may further increase due to rapid economic expansion and globalization [6]. 

The broad base of knowledge created by studying cancer cell helps to limit the progress of the disease. For many years, cancer was considered as a fatal disease and not curable and this creates a sense of fear and hopelessness in the patient’s mind, and the pain and side effects during chemotherapeutic treatment. So, to create a better and sustainable health care system and treatment, and to fulfill the medical needs of those with cancer and give them the highest life expectancy, scientific study in the field of cancer has tremendous importance [7,8].

**Figure 1:**
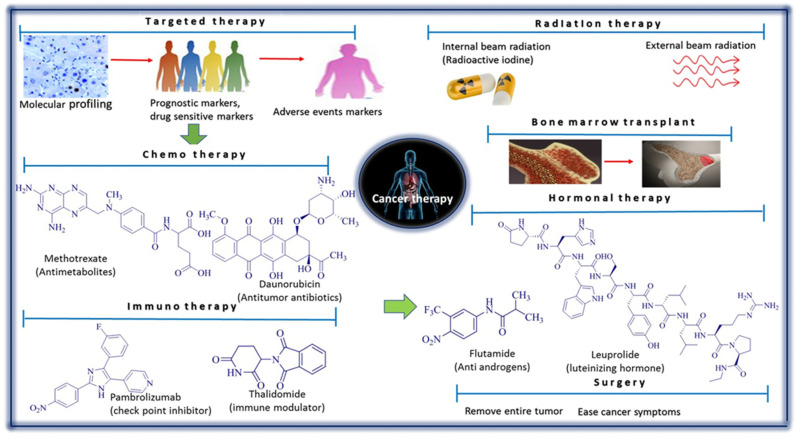
Major types of cancer treatment methods used by physicians and oncologists depending on the cancer type, stage, and severity. The targeted therapeutic method is using of drugs which identify and attack a specific type of cancer cell in the body. A molecular profiling system determines the abnormal gene manipulation inside cancer cell. Chemotherapy is uses to eradicate rapidly growing tumor inside the body. A chemotherapeutic drug Methotrexate slows the growth of cancer cells. Immunotherapy is based on the improving of the immune or natural defense system of the body for fighting the specific cancer. Thalidomide can be mentioned under the chemotherapeutic method for the treatment of bone marrow cancer. Radiation treatment uses beams of radiant energy like X-ray for killing of the cancer. Bone marrow transplant is replacing of the diseased bone marrow by the new bone marrow from the donor. Flutamide is an antiandrogen drug for the prevention of the testosterone from stimulating the cancer cells which leads to prostate cancer. Surgery is the method to completely remove the tumor or cancerous tissue.

The advancement of molecular and tumor biology changes the cancer treatment protocol that has been practiced for the past 15 years, based on histomorphology features and organ origin-based treatment [9]. Solving complicated biological and medical problems related to cancer is the priority to understand the causes of cancer, and how various factors help its growth and progression [10]. Figure 1 illustrates the various cancer treatments followed by doctors, physicians, and oncologists. Chemotherapy is one of the most common treatment methods, which uses one or more anticancer drugs to cure or prolong the life of the cancer patients. Immunotherapy is the artificial stimulation of the immune system against cancer cells, a targeted therapy interfering with the molecules in the cancer block and inhibiting cancer growth. Radiation therapy is the use of ionization radiation to kill malignant cells. Hormonal therapy inhibits the release of the hormones that facilitate the proliferation of cancerous cells. Bone marrow transplantation is replacing of damaged or diseased bone marrow. Surgery removes cancerous tumors [11,12]. In the traditional medicinal system, various medicinal plants have been reported to cure or treat infectious diseases, atherosclerosis, diabetes, cancer, etc. [13,14,15,16]. Traditional medicines are a first source of health care and traditional therapy throughout the world for around 80 –% of people who utilize medicinal plants [17,18,19].

**Figure 2:**
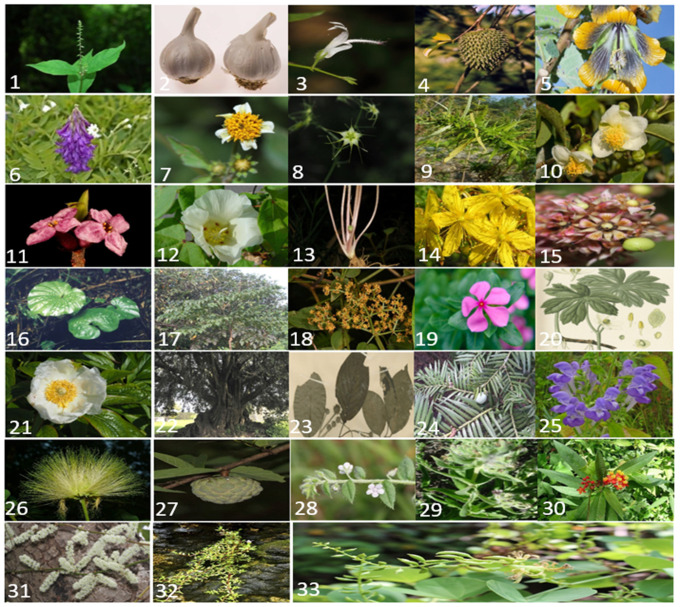
Photographs of medicinal plants with anticancer activity. (1) *Achyranthes aspera* (http://www.plantsoftheworldonline.org, accessed on 24 July 2022); (2) *Allium sativum* (http://www.plantsoftheworldonline.org, accessed on 24 July 2022); (3) *Andrographis paniculate* (http://www.plantsoftheworldonline.org, accessed on 24 July 2022); (4) *Annona muricata*(http://www.plantsoftheworldonline.org, accessed on 24 July 2022); (5) *Abutilon ranadei* (http://www.plantsoftheworldonline.org, accessed on 24 July 2022); (6) *Astralagus hedysarum* (https://www.bing.com/images/, accessed on 11 July 2021); (7) *Bidens pilosa*(http://www.plantsoftheworldonline.org, accessed on 24 July 2022); (8) *Bolbostemma paniculatum* (http://www.plantsoftheworldonline.org, accessed on 24 July 2022); (9) *Cannabis sativa* (http://www.plantsoftheworldonline.org, accessed on 24 July 2022); (10) *Camellia sinensis* (http://www.plantsoftheworldonline.org, accessed on 24 July 2022); (11) *Daphne mezereum* (http://www.plantsoftheworldonline.org, accessed on 24 July 2022); (12) *Gossypium hirustum* (https://th.bing.com/th/id/OIP.d_ accessed on 11 August 2021); (13) *Hydrocotyle asiatica* (http://www.plantsoftheworldonline.org, accessed on 24 July 2022); (14) *Hypericum perforatum* (http://www.plantsoftheworldonline.org, accessed on 24 July 2022); (15) *Mangifera indica* (http://www.plantsoftheworldonline.org, accessed on 24 July 2022); (16) *Nervelia fordii* (https://th.bing.com/th/id/OIP.og 1, accessed on 11 August 2021); (17) *Oroxylum indicum* (http://www.plantsoftheworldonline.org, accessed on 24 July 2022); (18) *Rubia cordifolia* (http://www.plantsoftheworldonline.org, accessed on 24 July 2022); (19) *Catharanthus roseus* (http://www.plantsoftheworldonline.org, accessed on 24 July 2022); (20) *Podophyllum peltatum* (http://www.plantsoftheworldonline.org, accessed on 24 July 2022); (21) *P. emodii* Wallich (http://www.plantsoftheworldonline.org, accessed on 24 July 2022); (22) *Taxus brevifolia* (http://www.plantsoftheworldonline.org, accessed on 24 July 2022); (23) Camptotheca acuminate (http://www.plantsoftheworldonline.org, accessed on 24 July 2022); (24) *Cephalo taxus harringtonia* var drupacea (http://www.plantsoftheworldonline.org, accessed on 24 July 2022); (25) *Centaurea ainetensis* (https://astraflowers.com/images/tinymce/386/Centaurea.jpg, accessed on 11 August 2021); (26) *Scutellaria Ocmulgee* (https://live.staticflickr.com/7111/7613258582_aac012ae12_b.jpg, accessed on 11 August 2021); (27) *Albizia lebbeck* (http://www.plantsoftheworldonline.org, accessed on 24 July 2022); (28) *Annona squamosa* (http://www.plantsoftheworldonline.org, accessed on 24 July 2022); (29) *Arnebia euchroma* (https://www.researchgate.net/profile/V_Tewari/publication/, accessed on 11 August 2021); (30) *Asclepias curassavica* (http://www.plantsoftheworldonline.org, accessed on 24 July 2022); (31) *Asaparagus racemosus* (http://www.plantsoftheworldonline.org, accessed on 24 July 2022); (32) *Bacopa monnieri* (http://www.plantsoftheworldonline.org, accessed on 24 July 2022); (33) *Bauhinia racemose* (http://www.plantsoftheworldonline.org, accessed on 24 July 2022).

For the advancement of cancer treatment and control of progression, much research has been done by scientists. Various cancer therapies have been used to cure or increase the life span of the patient. Different synthetic medicines have been used for the treatment of different cancers, but these medicines are also associated with several health risks to the patient. Therefore, the natural method of cancer treatment using plants or plant extracts has become a more popular method to cure cancer. In 1950, the investigation of anticancer phytochemicals from medicinal plants to treat cancer began with the isolation of alkaloids (vincristine and vinblastine) from Madagascar periwinkle, and *Catharanthus roseus* G. Don. and podophyllotoxin from *Podophyllum* species [20]. Several other anticancer agents, such as campothecin derivatives, homoharringtonine, vinca alkaloids, podophyllotoxin derivatives, and taxanes have been isolated from medicinal plants [21,22,23]. Some other medicinal plants reported for the treatment of cancer (Figure 2) include *Allium sativum (Allicin)* [24,25], *Camellia sinensis* [26,27], *Achyranthes aspera* [28,29], *Apis mellifera* [30,31], *Andrographis paniculate* [32], *Cannabis sativa* [33,34], *Astralagus hedysarum* [35,36], *Bidens Pilosa* [37,38], *Bolbostemma paniculatum* [39,40], *Centaurea ainetensis* [41,42], *Gossypium hirustum or Gossypium herbaceumalso* [43,44,45], *Hydrocotyle* [46,47,48], *Salvia miltiorrhiza* [49,50], *Hypericin perforatum* [51], *Annona muricata* [52,53], *Daphne mezereum* [54,55], *Picrorrhiza kurroa* [56,57], *Mangifera indica* [58,59], *Nervelia fordii* [10,36,60], *Rubia cordifolia* [61,62,63], *Silybum marianum* [64,65], *Scutellaria* [66,67,68], *Oroxylum indicum* [69,70], *Smilax china* [71], *Strychnos nuxvomica* [72,73,74], *Terminalia chebula* [75,76], *Vernonia amygdalina* [77,78,79], *Taraxacum officinale* [80,81], *Withania somnifera (Withanolides)* [82,83], *Brugmansia suaveolens* [84] *Zingiber officinale* [85,86,87], and *genus artemesia* [88]. This review highlights the important medicinal plants and their phytocompounds used for the treatment of cancer. Forty-five different medicinal plant species with their origins in India and with anticancer activity including against cancers of the colon, nasopharynx, kidney, Ehlrich ascites carcinoma, liver, prostate, lung, breast, and stomach are listed in Table 1. In Table 2 are listed the major phytochemicals extracted from the medicinal plants, such as vinca alkaloids, podophyllotoxin, taxanes, camptothecin, homoharringtonine, saponin, isoquinoline alkaloid, and shikonin phytocompound, with chemical structure and anticancer activity against breast cancer, lung cancer, lymphomas, testicular cancer, bronchial cancer, lung cancer, ovarian cancer, colorectal cancer, myelogenous leukemia cancer, chronic myelogenous leukemia cancer, cervical cancer, larynx cancer, and liver cancer.

**Figure 3:**
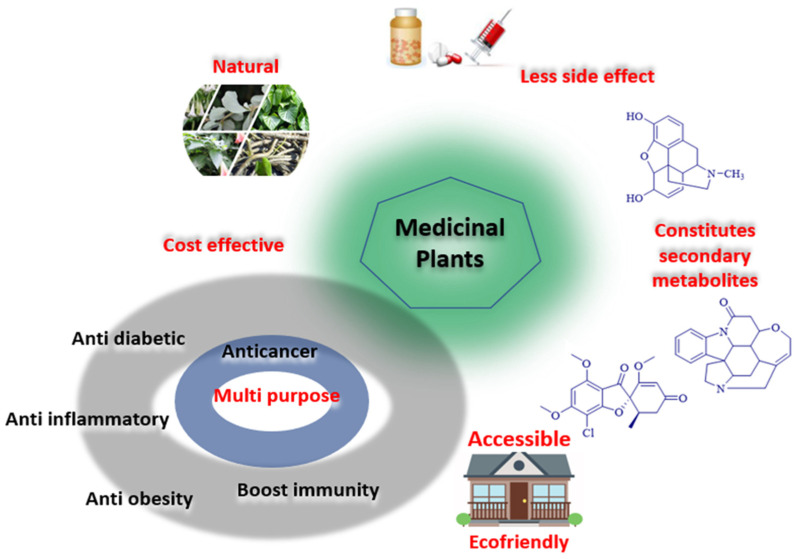
Advantages of using medicinal plants for the treatment of cancer. Synthetic drugs are manufactured in the factory using different chemicals, even though these drugs can cause side effects that are a danger to the patient. From this concept, various scientists have investigated a natural way of preparing drugs to treat cancer, and plants have become the main source due to their natural and non-harmful chemicals (phytochemicals), fewer side effects, easy accessibility, cost-effectiveness, and because chemicals can be obtained from one plant that have various functions such as antioxidant, antimicrobial, anticancer, etc.

Various reasons can be stated for the choice to use plants as a medicine (Figure 3) throughout history until the advent of modern medicine. The advantage of using medicinal plants for treating cancer over synthetic medication is due to their less side effects, high potency, less patient toxicity, affordability and ecofriendly. In addition of treating cancer, secondary metabolites also have anti-diabetic, anti-inflammatory, anti-obesity, and immune-boosting properties [138].

**Figure 4:**
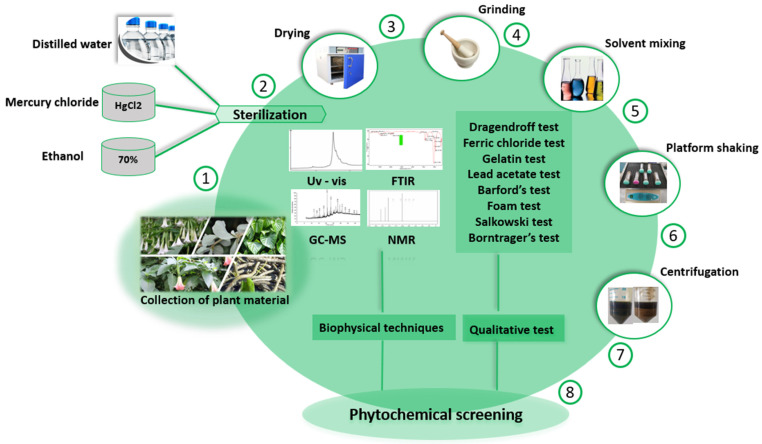
Scheme for analysis and characterization of phytochemicals. The collected plant material having medicinal properties undergoes different steps of preprocessing. The above figure illustrates the preparation of extract followed by phytochemical screening. The plant material collected from the field must be washed using tap water to remove dust, followed by treatment with disinfecting agent to kill any microorganism present on the surface of the plant. After treatment, the harvested plant part is dried in an incubator. After drying, the plant material is converted to a fine powder using a grinder. The powdered plant material can be used for the preparation of extracts using solvents of different polarities. Different methods such as sonication, Soxhlet, and maceration (cold/hot) of extractions can be used to prepare extracts.

The extraction of phytochemicals and analysis of anticancer properties of medicinal plants follows different steps (Figure 4). The parts of the medicinal plant needed to extract the phytochemical must be collected freshly and cleaned using distilled water, mercury chloride, 70% ethanol, and finally distilled water. The plant material is then dried in a hot air oven at 40–45 °C until ready for grinding. Then dried plant material is then made into a fine powder using a mortar and pestle or any mechanical or electrical grinder [139]. The phytochemicals can then be extracted from the dried power using a solvent of different polarity, ranging, for instance, from hexane, a non-polar solvent, to methanol, a polar solvent used to extract phytocompounds based on the principle of “like dissolves like”. This process can be done using rockers. Centrifugation or filtering of the slurry helps to separate the soluble phytocompounds from the insoluble material, then the solvent extract is dried at 40 °C for a further qualitative and quantitative analysis using a known concentration of the extract (µg/mL). Qualitative analysis of the phytochemicals uses a Mayer’s test or Wagner’s test to detect alkaloids, Ninhydrin test for aminoacids detection, Molish’s or Benedict’s test for carbohydrate detection, Borntrager’s and legal tests to detect the presence of glycosides, ferric chlorides test, gelatin, lead acetate, and alkaline reagent test for phenolic and tannins detection, Libermann–Burchard’s test for phytosterol’s detection, and Millon’s test or Biuret’s test to detect the presence of proteins. The biophysical analysis method is used to determine the phytochemical content qualitatively and quantitatively, gas chromatography is used to detect volatile phytocompounds by the principle of mobile phase and stationary phase, when the compounds separate according to their volatility, with highly volatile phytocompounds eluted out and detected. High-performance liquid chromatography (HPLC) also uses solvent as a mobile phase and a packed column as a stationary phase and uses 400 bars of pressure to elute the compound. High-performance thin layer chromatography (HPTLC) is the use of advanced thin layer chromatography that contains high-performance layers containing a sorbent of particles coated with 5–7 microns particle size with a layer thickness of 150–200 microns. UV spectroscopy is used to detect phytochemicals by passing the beam of light through the sample and determining the absorption, transmission, and reflectivity of the sample, and measures their wavelength by applying Beer–Lambert’s law and determining the concentration of the analyte solution. Nuclear magnetic resonance (NMR) spectroscopy determines the phytochemical structure using a one-dimensional method and the complicated phytochemical structures using a two-dimensional method [140]. Thin layer chromatography also uses the mobile and stationary phases to separate phytocompounds according to their relative solubility. Phytocompounds with higher affinity to the stationary phase move slowly and vice versa [141]. Fourier-transform infrared (FTIR) spectroscopy helps to determine the functional group of the extracted phytocompounds from the sample with the infrared range of 12,800~10 cm^−1^ [142].

Some medicinal plants effective against different types of cancers are discussed below.

*Achyranthes aspera*: *A. aspera* belongs to *Amaranthaceae* family and is found as a weed on the roadsides in India. The fractions from methanol extracts of stem bark such as saponin fractions alkaloid and non-alkaloid prevent EBV (Epstein–Barr virus) [143]. In in vitro study, the highest inhibitory activity is shown by non-alkaloid fractions containing nonpolar compounds [144], whereas during in vivo study the whole methanol extract shows cytotoxic activity against the mouse skin carcinogenesis in two-stage [29]. The *Achyranthes aspera* leaf DMSO extract has 100% cell growth inhibition. The direct cell count analysis method is used to check the cytotoxicity effect of the extract and shows a percentage proliferation reduction with values 24.9 ± 0.3 and 27.7 ± 0.3 percent at 200 µg/mL in 72 h and 24 h of medium replacement treatment, respectively [145].

*Allium sativum (Allicin)* or lasun, garlic: A plant used for medicinal purposes to treat numerous diseases. The cytotoxic effect of this plant is between 2–50 µg/mL as tested on the primary fibroblast, non-tumorigenic, a cell line which is permanent and obtained from kidney cells of baby hamster, and a Burkitt lymphoma origin of the tumor genic lymphoid cell line [146]. S-allyl cysteine, an organosulfur compound obtained from garlic, helps to destroy and delay transplantable and chemically induced tumors [147]. Two-hundred and fifty milligrams per kilogram of garlic has been administered in male Wistar rats thrice a week and shows the suppression of 4-nitroquinoline-1-oxide [148]. Four tumor cell lines of colorectal cancer cells (DLD-1), breast cancer cells (MDA-MB-231 and MCF-7), and lung carcinoma cells (SK-MES-1) were treated with different concentrations of *A. sativum* extracts (garlic hydroalcoholic extract and Welsh onion extract) between 10% and 0.156% extract in cell culture medium and between 2.5% and 0.156% concentration, respectively. The IC_50_ for both Allium extracts (garlic hydroalcoholic extract and Welsh onion extract) were higher than 1.25% extract (equivalent to more than 1 mg/mL). The IC_50_ value of *A. sativum* garlic extract showed a high effect on the human lung carcinoma cell line (SK-MES-1), accounting for 4.651%; whereas fewer effects were seen and accounted for 5.482% of the colorectal adenocarcinoma cell line (DLD-1), 6.131% for a breast cancer cell line (MCF-7), and 6.375% for an epithelial human breast cancer cell line (MDA-MB-231). The IC_50_ values of Welsh onion extract on the most sensitive cell line, colorectal adenocarcinoma cell line (DLD-1), were 2.124%, and less sensitive for an epithelial human breast cancer cell line (MDA-MB-231), 3.353% for a breast cancer cell line (MCF-7), and 5.819% for human lung carcinoma cell line [149]. The inhibitory effect of these extracts is dependent on the doses given and Allium and Allicin extracts’ inhibitory effect depends on the concentration of extract added to the cells. On the studied tumor cells, Allicin and allyl sulfides, which are bioactive compounds, had antitumoral and growth inhibition effects [150,151,152,153].

*Andrographis paniculate*: From this plant fourteen compounds have been identified during phytochemical identification in the ethanol extraction method of the aerial plant part. Labdane diterpenoids and flavonoids constituted a large amount of it. The isolate has the potential to inhibit tumor growth as investigated in different cell lines [154]. The dichloromethane fraction contains three variously active constituents that have cytotoxic and immune-stimulating activity [32]. However, it has side effect including headache, fatigue, gastric upset, and bitter taste and also induces malfunction of the liver if consumed in high amounts [36]. Ethanol extract of *A. paniculata* leaf has inhibition concentration (IC_50_) for IMR-32 (M2 subtype mRNA) and human colorectal adenoma–carcinoma (HT-29 cell lines) at 200 μg/mL, whereas other extracts have 50% inhibition effect at 250 μg/mL concentration for HT-29 cell lines. Anticancer activity of water, ethanol, and acetone extracts of *A. paniculata* leaves against HT-29 cancer cell lines had a 50% inhibition at 200 μg/mL concentration [155].

*Annona muricata*: This is a scientific name of Graviola, which contains acetogenins having huge medicinal importance that hinder the production of ATP (adenosine triphosphate) in human cells, and will have a significant impact in the eradication of cancer drugs. *Annona muricata* is extracted from the seeds, bark, fruit, and leaves. In addition, acetogenin has a chemotherapeutic ability against multiple drug-resistant cancers [156]. Some acetogenins are toxic for specific cancer cell lines, such as carcinoma tumors, prostatic adenocarcinoma, lung solid human breast cancer, human lymphoma, pancreatic carcinoma, multiple-drug resistant human breast adenocarcinoma, liver cancer, human lymphoma, and colonic adenocarcinoma [36]. The ethanolic extract of *A. muricata* was tested for anticancer activity against MDA and SKBR3 breast cancer cell lines using the MTT assay method. The anticancer property of water and ethanolic extracts of *A. muricata* against EACC (esophageal adenoid cystic carcinoma) was tested using Trypan blue-exclusion assay. An ethanolic leaves extract of *A. muricata* showed 32.9% inhibition of cell death at a concentration of 250 µg/mL and 100% maximum inhibition of cell death at a concentration of 750 µg/mL, even though the water– leaves extract of different concentrations had no inhibition effect on the cancer cell line. IC_50_ of ethanolic extracts was determined to be 335.85 µg/mL [53].

*Apis mellifera*: The honeybee or *Apis mellifera* (scientific name) has a protein to suppress apoptosis and enhance the primary cultured hepatocyte proliferation [157]. It also has a cytotoxicity capability in normal lymphocytes and human leukemia cell lines [158]. Surgical wounds coated with honey pre- and post-operatively on the cancer cell decrease the cancer tumor wound [159]. Ranging from 43.8 to 53.5 μg/mL IC_50_ value of crude dichloromethane and 41.3 to 52.4 μg/mL of hexane extract of *Apis mellifera propolis* had antiproliferative activity against colon adenocarcinoma (SW620), undifferentiated lung (Chaco), liver hepatoblastoma (Hep-G2), duet carcinoma (BT474), and gastric carcinoma (KATO-III) of human cancer cell lines. Cardanol and one cardol are the two main bioactive components isolated from *Apis mollifera*. In in vitro cytotoxicity and antiproliferation tests, IC_50_ values ranged from 10.8 to 29.3 μg/mL of cardanol in all five cancer cell lines whereas they were <3.13 to 5.97 μg/mL (6.82–13.0 μM) for the cardol in all five cancer cell lines. The cell death and cytotoxicity take place without DNA fragmentation of the cell [31].

*Astralagus hedysarum*: Contains a polysaccharide that has an antitumor activity which increases the third component complement (C3) of macrophages after two hours. When the injection increases by 5 times, the C3 positive macrophage proportion will be higher than after the first injection. Thus, the plant has cancer chemotherapy capability and immune potentiating action [160]. *Astralagus membranaceous* is another species of *astralagus that* repairs the impaired T cell mechanisms in the cancer patient. The antitumor effect of the plant has been studied and shown the antitumor immune activity of the host in both in vivo and in vitro [161]. Using various chromatography techniques, more than 155 phytocompounds have been extracted a few decades back and the phytocompounds identified using hydrogen nuclear magnetic resonance (H-NMR), carbon-13 nuclear magnetic resonance (13C-NMR), two-dimensional (2D-NMR) and high-resolution mass spectrometry (HR/MS) techniques [162,163,164]. HPS-1 phytocompound extracted from the roots of *Hedysarum* has the potential to suppress the proliferation of human gastric cancer cells (MGC-803) and inhibit the proliferation of human hepatocellular carcinoma (HEP-G2) (*p* < 0.05) at a concentration ranging from 50 to 400 μg/mL. Forty percent at 400 μg/mL is the proliferation inhibition ratio of the HPS-1 phytocompound. IC_50_ values of 10.69 μmol/L of compound 78 had an inhibitory effect on the human liver cancer cell line (HepG2) [165,166].

*Bidens Pilosa*: Contains phytochemicals terpenoids, polyacetylenes, phenylpropanoids, flavonoids, etc. The extraction of the plant and fractionation, isolation, and characterization yield the phenyl-1,3,5-heptatriyne, which reveals the toxicity of normal blood [37]. The antitumor effect of methanol, hexane, and chloroform plant extract and fractionation shows a positive result, though hexane extract shows a remarkable effect [38]. Petroleum ether extract of *Bidens Pilosa* had an inhibition effect of proliferation on a human epithelial cancer cell line (A549) with IC_50_ of 49.11 ± 2.72 μg/mL, a hepatoma cancer cell line (HepG-2), a human nasopharyngeal carcinoma cell line (CNE-2), and a murine tumor (B16) [167]. Triterpenes, the main constituents in petroleum ether extract of *Bidens Pilosa*, show antitumor activity both in vivo and in vitro [168]. Supercritical fluid extraction and hydro-ethanol maceration of *Bidens Pilosa* plant yields polyacetylenes phytocompound which has anticarcinoma activity and mainly kills breast carcinoma (MCF-7). The half maximal inhibitory concentration (IC_50_) value of supercritical fluid extraction (SFE) had IC_50_ = 437 (428–446) µg/mL in 24 h of incubation and lower to IC_50_ = 291 (282–299) µg/mL at 48 h. In MTT assay method, the IC_50_ value HCE showed 811 (795–828) µg/mL IC_50_ in 24 h of incubation whereas SFE had 437 (429–446) µg/mL of IC_50_ value [169].

*Bolbostemma paniculatum*: A compound called triterpenoid saponin tubeimoside-V was investigated after the extraction and fractionation of the plant that leads to isolation and characterization and revealed apoptotic clearance of glioblastoma cells and helped as antitumor chemotherapy [170]. Cytotoxic activity shown by tubeimosides such as tubeimodes-I and tubeimoside-II help DNA formation inhibition and lead to tumor cell phenotypical reverse transformation [171]. The phytocompounds isocucurbitacin B, cucurbitacin E, and 23,24-dihydrocucurbitacin E had strong anticancer activity against cervical cancer (HeLa) and human colorectal adenocarcinoma (HT-29) cell lines with values in the 0.93 to 9.73 μM IC_50_ range. Tubeimoside I, tubeimoside III, tubeimoside V, dexylosyltubeimoside III, and lobatoside C phytocompounds affected a human gastric cancer cell line (BGC-823), cervical cancer (HeLa), colorectal adenocarcinoma (HT-29), and breast carcinoma (MCF-7) with 1.30 to 15.64 μM IC_50_ values range. Tubeimoside I [40], the saponin extraction from *Bolbostemma paniculatum*, had anticancer activity by inhibiting the dissemination and reduction of p-PI3K, p-Akt, and p-mTOR protein expression levels of triple-negative breast cancer (MDA-MB-231) with IC_50_ value of 10 μg/mL at 48 h [172].

*Cannabis sativa*: Marijuana or cannabis sativa helps to eradicate tumor production and inhibit breast cancer cells [173]. Cannabinoids are an active constituent of cannabis sativa that has a palliative effect in cancer patients and has antitumor activity, stimulates appetite, and prevents pain, vomiting, and nausea [174]. The 48 h exposure of breast carcinoma (MCF-7) to dichloromethane (DCM) extracts of *C. sativa* samples with IC50 values < 50 μg/mL, the two DCM extracts of two *C. sativa* L. samples (DTP-06 and DTP-13) reduced extracellular vascular endothelial growth factor (VEGF) level significantly [175]. Cannabinoids tetrahydrocannabinol (Δ^9^-THC) and cannabidiol (CBD) are allowed in medication and legalized in the utilization of *Cannabis sativa* L for medical purposes. Terpenes or terpenoids, or their specific combinations, also contribute to carcinoma treatment and had a cytotoxic effect on colorectal carcinoma (Ht-29). The six different genotypes of *C. sativa constitute* cannabinoids and terpenes or terpenoids (linalool, limonene, α-terpineol, β-caryophyllene, α-humulene, *trans*-α-bergamotene, β-eudesmol, β-caryophyllene oxide, γ-eudesmol, guaiol, and α-bisabolol) that had anticarcinoma activity on hepatocarcinoma human cell lines (Hep-G2), colorectal carcinoma human cell lines (Caco-2), and colorectal carcinoma (Ht-29) are known as Conspiracy Kush, Jilly Bean, Jack Cleaner 2, Jack Skellington, Nordle, and Nurse Jackie. All extracts in the Nurse Jackie genotype extract had a significant cytotoxic effect on colorectal carcinoma (Ht-29). At the concentration of 8.48 ± 2.4–16.14 ± 0.07 μg/mL, the Jack Cleaner 2 extract had cytotoxic against hepatocarcinoma human cell lines (Hep-G2), and was positively selective for colorectal carcinoma human cell lines (Caco-2) and colorectal carcinoma (Ht-29) cell lines, whereas Jilly Bean showed 20.13 ± 3.05–49.88 ± 1.5 μg/mL high concentration, which was effective on each cell line, although Ht-29, Caco-2, and Hep-G2 hepatocarcinoma cancer cell lines were highly positively selective compared to healthy cell lines such as a primary cell line derived from the small intestine of a 3 to 4 mo gestation human fetus FHs 74 Int and medical research council cell strain 5 (MRC-5) [176].

*Centaurea ainetensis*: The crude extract of the plant can prevent the proliferation of colon cancer. The crude extract decreases the degree of tumors and the average size of deviant Aberrant Crypt. Isolation and characterization of a sesquiterpene lactonemolecule called Salograviolide-A (decreases the development of colon cancer cells) showed growth inhibition in the colon linked when the further fractionation of crude extract takes place. Salograviolide A has a cytotoxic effect against epidermal squamous cell carcinogens [41,42]. The ethanolic extract from the flowering part of *Centaurea ainetensis* with an IC_50_ value of 63.18 µg/mL had the highest cytotoxicity activity on a cervical cancer cell line (HeLa), adenocarcinoma human alveolar basal epithelial cells (A549) with 252.5 µg/mL IC_50_ value, and 69.27 µg/mL IC_50_ values of Daudi cells. It also showed lower cytotoxic activity on lung epithelial cell line (BEAS-2B cells) with an IC_50_ value of 75.25 µg/mL, when compared with the effects on the HeLa and Daudi (the most sensitive cell lines against the ethanolic extract) cancer cell lines [177].

*Camellia sinensis*: Constitutes EGCG, which is a polyphenol Epigallocatechin-3-gallate that helps to limit the metastasizing and invasion of human oral and colon cancer by decreasing gelatinase A, urokinase plasminogen activator, and matrix metalloproteinase 9 activator [178]. Epigallocatechin-3-gallatecan also inhibits hepatocellular carcinoma cell lines and other cancer cell lines by halting the progression through the cell cycle arrest [179]. EGCG prevents the development of cancer cells including human rectal, HCA-7, and HT-29 a, and in OVCA (ovarian carcinoma cell lines) and HEY [180,181,182]. A hot water extract of *Camellia Sinensis* had antiproliferative activity against a human colon cancer cell line (HT-29) with IC_50_ value of 86.68 ± 0.73 µg/mL. The phytoconstituents from *the camellia* sinensis plant and also its synthesized compounds had tremendous anticarcinoma activity against esophageal cancer, lung cancer, stomach cancer, liver cancer, and skin cancer [183,184,185]. Due to the presence of polyphenols, the plant exhibits antiproliferative activity against colonic tumorigenesis [186,187].

*Daphne mezereum*: *Daphne mezereum*, an anti-leukemic compound, in hydro alcohol extract has an antileukemic effect against lymphocytic leukemia investigated in mice. Mezerein was obtained after a fractionation study on the extract by isolation and characterization [188]. Dichloromethane and n-hexane extracts of the aerial part of *Daphne mezereum* had anticancer activity against breast carcinoma (MCF-7) with an IC_50_ value of 6.08 and 15.0 μg/mL of the respective extracts. Antiproliferative activity of dichloromethane extract on the metastatic renal adenocarcinoma (ACHN) with an IC50 of 16.17 μg/mL, and both n-hexane and dichloromethane extracts, acted against skin malignant melanoma cancer cell line (C32) cells with 16.55 and 19.88 μg/mL IC_50_ values. From *Daphne* species, the *D. striata* also showed IC_50_ values < 30 μg/mL against breast carcinoma (MCF-7), adenocarcinoma human alveolar basal epithelial cells (A549), and a human prostate cancer cell line (LNCaP), which was the most sensitive to dichloromethane extract at IC_50_ of 13.98 μg/mL, renal adenocarcinoma (ACHN), a skin malignant melanoma cancer cell line (C32), and was most sensitive in the application of methanol extract with the value of IC_50_ 15.51 μg/mL and 142BR cell [189].

*Gossypium hirustum*: Cottonseed oil or gossypol has medicinal properties to treat endometrial or ovarian metastatic cancer, act as a male contraceptive, and treat HIV. In mitochondrial and cytosolic enzyme systems which are a factor for tumor cell growth including endometrial, brain, prostate, colon, lung, melanoma, and adrenocortical cancer, gossypol showed anti-tumor activity [190,191,192]. Because of its potential toxicity, there is no specific dosage of administration to treat and for self-medication [193]. The methanolic extract of the *Gossypium hirsutism* bolls showed anticancer activity against human breast cancer (MCF-7) cells with 2.72 IC_50_ values, human lung cancer (A549) cells with 4.02 IC_50_ values, glioblastoma cells (U87) with 4.22 g/mL IC_50_ values, and human hepatocellular carcinoma (HepG2) [50]. The phytochemicals that constitute in *Gossypium hirustum* plant are cardiac glycosides, phenols, tannins, saponins, alkaloids, flavonoids, terpenoids, and protein [194]. A natural phenol-derived phytocompound, gossypol showed antiproliferative activity against gliomas, mammary carcinomas, adrenal, prostate, endometriosis, and uterine myoma [92,195].

*Hydrocotyle asiatica*: Known by its common names Gotu kola, brahmi, and scientific name as *Hydrocotyle asiatica*. It has antitumor and cytotoxicity activity. The dried leaves given at 600 mg thrice a day increased the life expectancy of a cancer-tumor patient as investigated in mice [196]. Clothing and sunscreen are needed to prevent sunburn because of effects of increased photosensitivity [197]. Cytotoxic activity has been seen against rat glioma cell lines and human breast cancer melanoma of mice during the injection of aqueous extract of the Gotu kola leave [198]. *C. Asiatica* crude extract and the pure fraction helped to hinder the development of ascites and harden tumors, increasing the life expectancy of the tumor-diseased mice. Cytotoxic effects and antitumor activity include the direct action on DNA synthesis [199]. The aqueous extract of *Cantella Asiatica* leaves had significant activity against mouse melanoma (B_16_F_1_) with IC_50_ values of 698.0 μg/mL, human breast cancer (MDA MB-231) with IC_50_ values of 648.0 μg/mL, and rat glioma (C_6_) with IC_50_ values of 1000.0 μg/mL in a cancer cell line [200].

*Hypericum perforatum*: Constitutes hypericin that is isolated and characterized by the plant, which limited and hindered the glioma cell line’s growth and led to glioma cell death (linked to protein kinase inhibition). Hypericin contains a compound called napthodianthrones. The effect of hypericin against glioma cells was higher compared to that of tamoxifen [201,202]. The main advantage of the *Hypericum perforatum* is the component it constitutes, which prevents serotonin reabsorption and thus decreases cell development on various cell lines [203]. The dimethylsulphoxide (DMSO) extract of *H. perforatum* constitutes a phytocompound phenol that inhibits the proliferation of human prostate cancer cells (PC-3) with an IC50 value of 0.21% [204]. The *Hypericum perforatum* 1–11 ethanol and water extract showed anticancer activity against a human cervical cancer cell line (HeLa), a human breast cancer cell line (HCC-1937), a human osteosarcoma cell line (U2OS), and one non-tumoral cell line, which was a human lung fibroblast cell line (MRC-5), whereas the extract of *Hypericum perforatum* 2 showed a high effect on a HCC-1937, cancerous tumor HeLa, MRC-5, and U2OS with IC_50_ values of 8.14 ± 0.54, 14.48 ± 1.15, 10.70 ± 0.31 μg/mL and 9.174 ± 1.17, respectively, without selectivity on either cancer or tumor cells, whereas with higher selectivity *Hypericum perforatum* 3 ethanol extract inhibited cell proliferation of HCC-1937 breast cancer cells [205].

*Mangifera indica*: This is used for nutritional purposes in various countries including Cuba, considering reducing nutritional and environmental risks, and has antioxidant activity by scavenging free radicals [206]. In cancer patients, it has shown the advancement in their life quality in ethnobotanical studies [207]. As it is used as a nutritional supplement, mango shows an immune modulation effect in cell lines, its constituent microelement polyphenols, terpenoids, fatty acids microelements, and steroids having antioxidant activity [208]. Mango leaf extract at 200 μg/mL showed a cytotoxicity activity against human cancer cell lines; ductal carcinoma (BT474, ATCC HTB20), bronchogenic carcinoma (Chago K-1, ATCC HTB-168TB), liver hepatoblastoma (Hep-G2, ATCC HB8065), gastric carcinoma (Kato-III, ATCC HTB103), and colon adenocarcinoma (SW 620, ATCC CCL227) with IC_50_ values > 200 μg/mL, even though mangiferin does not have any toxicity activity against all the cancer cell lines mentioned above [58,209,210].

*Nervelia fordii*: Using a mice model, ethyl acetate and petroleum ether extract, *Nervelia fordii* showed anticancer activity as demonstrated on S-180 mice and H-22 mice models and showed high anticancer activity, also increasing the life span of the cancer carrier. This gives the conclusion that *Nervelia fordii* as an anticancer agent also needs further research and investigation for isolation and identification of the major active components [211]. It also showed cytotoxic activity against human breast carcinoma (MCF-7), human liver carcinoma (HepG2), and human colon carcinoma cells [62,141].

*Oroxylum Indicum*: The famous Indian medicinally utilized plant has cytotoxic activity that is 95% of that of ethanol extracts on hep cell lines with 0.05% concentration [212]. The antitumor effect of flavonoid baicalein inhibits the multiplication of HL-60 up to 50% at 25–30 µm concentration [213]. The methanolic plant extract can prevent the mutagenesis of tryptophan-P-1 in Ames testing [214]. The fraction constitutes nitrosated that has cell proliferative, and a genotoxic activity administered 0.25–2 g/kg body weight, in rat stomach at the pyloric mucosa in vivo [215]; it also showed toxicity activity on tumor cell lines examined in half inhibitory concentration [216]. Methanolic and aqueous extracts had a huge cytotoxicity activity on the cancer cell line, a greater cytotoxic ability has been shown by the methanolic extract, but both extracts can protect DNA against oxidative stress [217]. The ethanolic root extract of *Oroxylum indicum* had significant toxicity on murine melanoma (B-16), human colon carcinoma (HCT-8), lymphoblastic cells (CEM), and leukemia (HL-60) and tumor cell lines, with an IC_50_ of 19.6 µg/mL for lymphoblastic cells, 14.2 µg/mL for leukemia, 17.2 µg/mL for murine melanoma, and 32.5 µg/mL for human colon carcinoma [218].

*Rubia cordifolia*: The antitumor activity is via the chemicals hexapeptides and quinones binding with eukaryotic 80S ribosomes, and this leads to translocation of peptidyl-tRNA and binding inhibition of aminoacyl-tRNA, needed for protein synthesis [20,219,220]. *Rubia cordifolia* roots chloroform extract constitutes the mollugin phytochemical, which is beneficial for inhibition of mast cells’ degranulation, passive cutaneous anaphylaxis prevention, and leukemia prevention [221]. The cytotoxic chemical constituents extracted from the roots and I and II DNA topoisomerase inhibition capability of the root extract were examined, and the activity of topoisomerases I and II inhibition level determined using the supercoiled PBR 322 plasmids’ DNA relaxation. For breast carcinoma, colon carcinoma cytotoxic determination, and liver carcinoma (HepG2) cell lines the MTT (tetrazolium-based colorimetric) assay is applicable. A total of seven different compounds were extracted that are useful for cytotoxic activity [222]. Cytotoxicity was confirmed of the methanolic root extract of *Rubia cordifolia* on HeLa cell with IC_50_ values of 23.12 μg/mL, pet ether fraction with IC_50_ values of 38.13 μg/mL, and dichloromethane fraction with IC_50_ values of 48.87 μg/mL. The root extract of *Rubia cordifolia* showed cytotoxicity activity with IC_50_ values of 8.57 μg/mL of methanol fraction, 10.51 μg/mL of pet ether fraction, and 16.72 μg/mL of dichloromethane fraction against a human leukemia cancer cell line (HL60). The moderate cytotoxic activity was shown with IC50 values of 27.33 μg/mL of methanol fraction, 35.44 μg/mL of pet-ether fraction, and 41.59 μg/mL of dichloromethane fraction against hematopoietic cell line (U937) [34].

*Scutellaria*: Constitutes biologically active flavonoids such as baicalein, wogonin, chrysin, apigenin, and scutellarein. The flavonoid of the plant species shows antitumor activity. The leaf extract of *Scutellaria Ocmulgee*, *Scutellaria angulosa*, *Scutellaria scandens*, and *Scutellaria integrifolia* shows anticancer effect [223]. *Scutellaria barbata* has shown antitumor activity as investigated on the cell line of human lung cancer. A novel anticancer agent, *Scutellaria baicalensis*, had a significant antiproliferative activity with the IC_50_ values 1.1, 0.9, 0.52, 0.82, 1.1, 1.5, 1.0, and 1.2 mg/mL against hepatic carcinoma (HepG2), human breast carcinoma (MCF-7), a human prostate cancer cell line (PC-3), a human prostate cell line from xenograft (LNCaP), colon carcinoma (KM-12), a human colon cancer cell line (HCT-15), epithelial carcinoma (KB), and human squamous cell carcinoma (SCC-25), respectively [224]. Ethanolic extract of *Scutellaria barbata* species decreased A549 cell growth, with a half inhibitory concentration at 0.21 mg/mL [224]. Anti-breast cancer, signal transduction, apoptosis, invasive potential, transcriptional regulation cell proliferation, and angiogenesis were performed by Tanshinone IIA extracted from the herb *Scutellaria litwinowii*, *S.baicalensis* species, etc., which showed anticancer activity in various studies [225,226].

Orchid Plants: A recent review of orchid plants including *Dendrobium chrysotoxum*, *Dendrobium officinale Caulis*, *Ephemeranthalon chophylla L*, *Eulophobia macrobulbon*, *Eulophia nuda*, *Gastrodia elata*, *Pholidota cantonensis*, *Spiranthes australis*, *Vanda cristata*, *Dendrobium transparens*, *Dendrobium formosum*, *Dendrobium nobile*, *Dendrobium moniliforme*, *Anoectochillus formosanus*, *Bulbophyllum odoratissimum*, etc., possess good anticancer properties on different types of cancer [227].

## 2. Major Anticancer Agents Obtained from Medicinal Plants

WHO recommends cancer is the leading cause of death worldwide, with nearly 10 million deaths predicted in 2020(1) [228]. The bioactivities of phytocompounds for various health benefits have been studied for decades [229]. Synthetic drugs are being replaced by phytocompounds which have great advantages due to their effects on a wide range of target cells with lower cell cytotoxicity effects or side effects compared to synthetic anticancer compounds, which are a single-target effect for prevention and treatment of carcinoma. Various medicinal plants and their nanoparticles have anticancer activity, namely *Murraya* *koenigii* leaf extract ZnO nanoparticlets [230,231]. Most modern drugs used to treat cancer originate from various medicinal plants; 50% of the anticancer drugs originate from medicinal plants. In developing countries, more than 80% of people use medicinal plants as traditional medicinal therapy and 60% of cancer patients use herbal treatment as an option to cure cancer [232,233]. Currently, for high-risk breast cancer patients, tamoxifen and related compounds such as raloxifene are prescribed [234]. The phytocompounds most studied in different research papers for the treatment of cancer (anti-proliferative activity) are curcumin, polyphenols, Withaferin A (WFA), a triterpenoid, celastrol, and berry bioactives [1,15]. Vinca alkaloids, podophyllotoxin, taxanes, campothecin, homoharringtonine, saponin, isoquinoline, shatavarine IV, stigamsterola, calotropin, and shikonin phytocompounds are discussed as follows.

### 2.1. Vinca Alkaloids

According to the declaration of WHO Model List of Essential Medicines on 8 December 2016, from the plant kingdom the first antimitotic agent used in the pharmaceuticals and drugs market and known to be highly effective in the treatment and utilized medicines was Vinca alkaloids. Vinblastine, vinca alkaloids, and leurocristine extracted from *Catharanthus roseus* were the first drugs utilized for cancer treatment purposes. These drugs were extracted from the Catharanthus roseus plant species during an investigation for the development of oral hypoglycemic agents, even though the extracts reduced white blood cell count and caused bone marrow depression in rats instead of the oral hypoglycemic treatment effect. The extracts obtained from this plant increase the life expectancy of mice treated with transplantable lymphocytic leukemia. Vindesine (VDS) and vinorelbine (VRLB) are the semi-synthetic analogs of vinca alkaloids, useful to treat various cancers by mixing with other chemotherapeutic drugs. Vinblastine is used to treat Kaposi’s sarcoma, breast cancer, leukemia cancer, testicular cancer, lung cancers, and lymphomas [235]. Vincristine is used to treat leukemia, and childhood acute lymphocytic leukemia [27].

### 2.2. Podophyllotoxin Derivatives

The derivatives of podophyllotoxin (PTOX) etoposide [236], teniposide [237], and etoposide phosphate [238], are used for anticancer chemotherapy that is extracted from *Podophyllum peltatum* L. (Berberidaceae) and *Podophyllum emodi* Wall. (syn. *P. hexandrum*). PTOX is an aryltetralin-lignan with strong cytotoxic activity [239]. The podophyllotoxin derivatives have antiproliferative activity against germ cell tumors and small cell and non-small cell lung cancers. 4β-aminoalkyl-4′-*O*-demethyl-4-desoxypodophyllotoxin, TOP-53, is a podophyllotoxin derivative with antitumor activity and anticancer activity against lung cancer and lung metastatic cancer. The cytotoxicity activity of TOP-53 was determined using IC_50_ and showed 0.016–0.37 µg/mL against murine tumors and 0.26–8.9 µg/mL against human non-small cell lung cancer (NSCLC) cell lines. TOP-53 podophyllotoxin derivative is also potent in antitumor activity for lung localized tumors and metastatic tumors in the lungs [240].

These derivatives prevent the polymerization of tubulin and thereby could induce cell cycle arrest at mitosis and inhibit the formation of the mitotic-spindles microtubules. The therapeutic use of a plant species of the *Podophyllum peltatum* Linn., *P. emodii* Wallich family Podophyllaceae, has been used for the treatment of warts and skin cancer. Native Americans used topical administration of alcoholic extract of dried roots of *Podophyllum peltatum* for the treatment of warts by its topical administration in the 1940s. The first isolated major cytotoxic therapeutic component was podophyllotoxins in the 1880s. During the development of spectroscopic techniques, the exact structure of podophyllotoxins was determined in the 1950s. Lignans, which are almost like podophyllotoxins, were also applied during clinical trials, but the effect was unsatisfactory concerning high cytotoxicity and absence of effectiveness. Later on between the 1960s and 1970s, Teniposide (C_32_H_32_O_13_S) and Etoposide (C_29_H_32_O_13_) were discovered and used as clinical agents to treat testicular, bronchial, and lymphomas cancer, as reported by Sandoz Laboratories in Switzerland [241].

### 2.3. Taxanes

The first isolated compound from *Taxus brevifolia* Nutt. (Taxaceae) bark was taxol or Paclitaxel. Various parts of *Taxus* species, such as *T. canadensis* Marshall, *T. baccata* L., and *T. brevifolia*, have been used for anticancer activity, for instance for the treatment of ovarian and breast cancers [114]. This has led to substantial demand for it. In the ancient Indian holistic and natural medicine called Ayurveda, the leaves of *T. baccata* were used in the treatment of cancer. Taxanes from *T. wallichiana* plant species have anti-inflammatory, analgesic, antipyretic, antiallergic, immunomodulatory, anticonvulsant, anti-conceptive, anti-osteoporotic, antiplatelet, antifungal, and antibacterial activities, as well as antispasmodic effects [242,243,244]. A *Taxus* species constituent, paclitaxel, is found in the leaves. Baccatins exist in high amounts and are converted to paclitaxel and active paclitaxel analogs such as docetaxel or Taxotere, which are a significant source and major category of the drugs, and are utilized to treat Kaposi sarcoma, lung cancer, ovarian and breast cancer. Paclitaxel also has the potential to treat non-cancerous diseases, such as rheumatoid arthritis, psoriasis, and multiple sclerosis. Breast cancer is mainly treated using a semisynthetic derivative of docetaxel. The effectiveness of the docetaxel anticancer agent was analyzed statistically by developing a clinical trial of more than one dozen taxanes analogues. The National Cancer Institute (NCI) recorded 2069 cancer clinical trials in July 2004 and stated that 105 with docetaxel (Taxotere), 10 with miscellaneous taxanes, 134 with Taxol (paclitaxel), and 23 taxanes, and in total around 248 listed as taxanes-derived drugs, are in preclinical development in single or combined with other anticancer agents [17]. The cytotoxicity effects of paclitataxel and docetaxel on a lymphoblastoid cell line were assessed in half maximal cell growth (IC_50_) to indicate the drug sensitivity as a phenotype and shown to be 3.98–21.36 nmol/L for paclitaxel and 1.54–13.32 nmol/L for docetaxel in the IC_50_ range [30].

### 2.4. Camptothecin Derivatives

For the first time, In the early 1960’s a phytochemical called camptothecin was extracted from a Chinese ornamental tree called *Camptotheca acuminata Decne (Nyssaceae)* species and used as an anticancer agent. This shows the advancements in anticancer drug development. An extract camptothecin from *Camptotheca acuminata species* showed high anti-tumor and anticancer activity out of 1000 different plant extracts tested for the same activities. This is considered the unique character of the *Camptotheca acuminata plant* species. The active chemicals isolated from it were identified as camptothecin, declared in the 1970s by the NCI (National Cancer Institute) as a candidate for clinical trials However, it displayed a flaw in bladder toxicity and was no longer in use. SmithKline Beecham (now Glaxo SmithKline) develops Topotecan (Hycamtin) and effective camptothecin derivatives, and Japanese company developed Irinotecan, Yakult Honsha, which are more effective than camptothecin. Irinotecan is utilized to treat colorectal cancers, whereas whereas lung and ovarian cancer are treated by topotecan [52]. TDP1 (Tyrosyl-DNA phosphodiesterase 1) inhibitors have a moderate inhibitory effect with IC_50_ which is the concentration of a compound required to reduce the enzyme activity by 50% in the concentration range of 0.4–100 µm [245,246,247]. A natural quinoline, camptothecin (CPT) has anticancer activity and inhibits Topoisomerases 1 [98]; it is also used as a chemotherapeutic drug to treat tumors and metastatic colorectal cancer and is known as a bioavailable derivative of irinotecan [248]. Topotecan is also a CPT derivative that treats ovarian cancer and small cell lung cancer [249]. TDP1 helps to prevent the DNA damage caused by Top1 inhibitors, hence TDP1 is responsible for drug resistance of some cancers. TDP1 activity and the percentage of non-small-cell lung cancer tumor cells in human tissue have a positive correlation [250]. The synergistic effect of TDP1 and TOP1 inhibitors is expected to increase treatment or decrease the traditional drug dose [251].

### 2.5. Homoharringtonine

*Cephalo taxus harringtonia* var *drupacea* (Cephalotaxaceae) is a Chinese tree by which semisynthetic cephataxine homoharringtonine was originally obtained and clinically used. Elliptinium is extracted from the Apocynaceae, a family containing *Bleekeri avitensis*, a medicinal plant from Fiji having anticancer activity. Chronic myelogenous leukemia treatment and myelogenous leukemia in China are achieved through the mixture of homoharringtonine (HHT) and harringtonine. For effective treatment of leukemias, purified homoharringtonine has been used because some cancers are drug-resistant. Breast cancer has been treated using Elliptinium in France [252]. HHT possesses antitumor activity. For instance, the administration of HHT 25 mg/kg inhibits the growth of tumor volume in the same way as in concentration of cisplatin [253]. In addition, cisplatin also possesses body weight reduction ability whereas HHT lacks this effect. From the experiment of a lung cancer xenograft mouse model, the effects of 10 mg/kg cisplatin administration and 15 mg/kg and 25 mg/kg HHT on tumors were 69.6%, 40.5%, and 74.6% respectively. From this, we can conclude HHT is an effective drug to treat lung cancer [254]. Omacetaxine mepesuccinate is a drug mainly used to treat breast cancer and leukemia. This drug works by blocking the synthesis in the peptidyl transferase center and leads to cell apoptosis [255].

### 2.6. Saponin Extract from Albizia Lebbeck Plant

*Albizia lebbeck* is a fast-growing deciduous and dispersed umbrella-shaped plant with a thin foliage crown with smooth, grayish-brown and fissured bark, found in Bangladesh, Africa, India, Australia, and subtropical and tropical Asia [256]. In traditional medicine, the *Albizia lebbeck* plant parts such as the leaves and pods part were utilized in cancer prevention. In addition, seeds, bark, pods, and leaves were applied for cytotoxic activity in colon, cervical, hepatic, breast, and larynx cancer [104]. Parts of *Albizia lebbeck* such as flowers, roots, bark, and seeds are utilized for the treatment of edema, poisoning, bone fracture [257,258], arthritis [259], skin disease, cough, and cold, wound healing, pruritis [53], malaria [258], abscess, abdominal tumors, leprosy, boils, and gonorrhea [260] in traditional medicine. Traditionally, the leaves of *A. lebbeck* were used for cancer treatment [261], and the pods also showed anticancer activity [168]. The poisonous and medicinal plants of east and South Africa declared *A. lebbeck* uses for its anticancer activity [262]. Stem bark methanolic extract of *A. lebbeck* has a cytotoxic effect against cervical carcinoma, larynx carcinoma, hepatocarcinoma, breast carcinoma cell lines, and colon carcinoma [263]. A saponins area secondary metabolite of glycosidic nature shows an anticancer effect [264]. Programmed cell death is caused by an enzyme called caspases which is from a family of proteases that activate and influence the executioner mode of apoptosis [104,265].

### 2.7. Isoquinoline Alkaloid Extract from Annona squamosa

*Annona squamosa* or custard apple, a small green tree, 6–8 m tall, is found specifically in deciduous forests. The medical applications are constipation, dysentery, antibacterial infection, epilepsy, dysuria, cardiac problems, hemorrhage, abortifacient properties, ulcers, fever, antifertility, antitumor, and worm infection treatments [266,267,268]. Constitutes a compound of acetogenins having anti-microbial, anti-neoplastic, pesticidal, parasiticidal, and parasiticidal effects [201,269]. Squamostatin and squamocin extracted from *A*. *squamosa* seeds compounds of acetogenins show a cytotoxic effect [270,271]. By the activation of caspase 3, squamocin prevents human leukemia cell line proliferation and leads to apoptosis. Another part of acetogenin called ascimicin can inhibit and is cytotoxic to 9KB, A549, HT-29, and 9ASK tumor cells [213]. To treat chronic diseases like such as skin complaints, insect bites, and cancerous tumors, all parts of *A. squamosal* were used in traditional medicine [43,49,272,273]. The phytochemicals existing in the leaves are anti-ulcer, anti-diabetic, anti-fungal, anti-inflammatory, anti-depressant, and antimicrobial [274,275,276,277,278,279]. The chemical compounds constituted in *Annona squamosa* are phenolic compounds, terpenoids, alkaloids, flavonoids, glycoside, saponin, and steroids which are all-natural products [215,280,281]. The alkaloids obtained from the aerial part showed anticancer activity in 0.01 to 100 µg/mL concentration ranges on liver, breast, and colon cancer cell lines. Isoquinoline alkaloid extract possesses a high anticancer activity against colon cancer cells (HCT116) and human breast cancer cells (MCF-7) [107].

### 2.8. Shikonin and its Derivatives

Traditionally, for anti-inflammatory and antimicrobial effects, and as a tonic for chronic diseases, remitting cough and cold purposes, *Arnebia euchroma* liquid extract is mixed with bees’ wax [282,283]. Phytochemicals constituted in the *Arnebia euchroma* which have great importance in anti-immune deficiency, anti-microbial and anticancer activity are arnebin-7, acetyl-shikonin, isovaleryl-shikonin, shikonincoumarins, B-hydroxy-isovaleryl-shikonin, deoxy-shikonin, β,β-di-methylacryl-shikonin, iso-butyryi-shikonin, stigma sterol, arnebinone, and isobutyl-shikonin [284,285,286,287,288]. A secondary metabolite of *Arnebia euchroma* called shikonin, found mainly in the root, prevents a compound that malfunctions and deletes the process of action in the cell, rapidly causing carcinomas [289]. Researchers also investigated some derivatives of the Sh+ikonin component and their cytotoxic effect and antioxidant activity [290,291]. From the plant species the methanol extracted has antioxidant and antimicrobial effects, and hydroxyl radicals for DNA damage protection [292]. The phytochemicals existing in the *Arnebiaeu chroma* plant are utilized for treating carcinogenic diseases. The phytochemicals utilized for treatment are acetyl-shikonin, teracryl-shikonin, and β,β-dimethylacryl-shikonin [18,200,293,294]. A phytochemical compound shikonin is utilized for various activities such as analgesic, anti-tumor, anti-fungal, anti-bacterial, wound healing, anti-inflammatory, anti-diabetic, anti-pyretic, and chemo-preventive [234,251,295,296,297]. For the preparation of various drugs effective inwith anti-inflammatory, anti–HIV, anti-microbial and anticancer effects, isovaleryl-alkannin, acetyl-shikonin, benzoquinone, shikonin-angelate, naphthoquinone, deoxy-shikonin, arnebin-5, arnebin-6, and alkannin are utilized from the phytochemical shikonin. The roots of *Arnebia euchroma* also constitute a dimeric naphthoquinone compound called Shikometabolin H, epoxyarnebinol, and 2,3-secodiplopterol dioic acid helps to reduce the STAT3 transcriptions, the activators of human carcinogenic cells, and increase the antitumor immunity [298,299].

### 2.9. Calotropin in Asclepias curassavica

A plant species *Asclepias curassavica* constitutes a wide variety of biologically active compounds such as flavonol glycosides, carbohydrates, triterpenes flavonols, cardenolides, amino acids, etc. The chemical called cardenolides has the constitutents calotropin, coroglaucigenin, calactinasclepin, asclepain CI, asclepiadin CII, curassavogenin, asclepogenin, calotropagenin, uzarin, uzarigenin, uscharidin, corotoxigenin, uscharidin, calotroposide, kidjolanin, clepogenin, and desglucouzarin, which are applicable for pharmacological purposes such as anticancer, antipyretic, analgesic, antimicrobial, cardiovascular, and many other pharmacological activities [300]. Calotropin (a cardiac glycoside), an alcoholic extract of *Asclepias curassavica* species, has a cytotoxic effect against nasopharynx carcinoma cells. A pronounced cytotoxicity activity against four different types of cancer cells was shown by cardenoliedes phytocompounds extracted from the aerial and root part of *Asclepias curassavica.* Significant cytotoxic activity was shown by asclepin and 12 beta-hydroxycalotropin (a cardenolide) had a strong cytotoxic effect against HepG2 and Raji cell lines [145,218,299].

### 2.10. Shatavarin IV in Asparagus racemosus

Compounds having anticancer activity include terpenoids, lignans, alkaloids, and flavonoids. Terpenoids (steroids) are the major group and widely applicable in chemotherapy cancer treatment, e.g., Taxol can be mentioned [301]. Steroidal saponin with few steroids and their glucosides, triterpenoids, alkaloids, and flavonoids exist in *Asaparagus racemosus* species [302]. Shatavarin I to X (shatavarins) are the major steroidal glucosides or saponins extracted from the root [132,303]. The non-polar and polar extracts from the total extracts and their formulation are capable of immune–pharmacological activity in cancer chemotherapy [304]. 7,12-dimethylbenzanthracene (DMBA)-induced mammary carcinogenesis can be inhibited by the *Asparagus racemosus* plant species extract, as investigated in rats. The compound shatavarin IV (84.69 %) with its fraction, codedAR-2B containing 5.05% shatavarin IV, is capable of cytotoxicity. Shatavarin IV from shatavarin’s rich fraction has a tremendous anticancer effect in vivo and in vitro [305].

### 2.11. Stigmasterol in Bacopa monnieri Linn

The plant *Bacopa monnieri* constitutes bacosides A and B, alkaloids, namely herpestine and brahmana, tetracyclic triterpenoid saponins, flavonoids, hersaponin, [306], triterpenes such as bacosine [307], and sterols like bacosterol [308]. A natural product, phytoserols extracted from the aerial part of the plant species *Bacopa monnieri* have anticancer activity [309]. The activity of stigmasterol tested on the growth of murine models of cancer, which becomes transplantable by decreasing the viable cell count, a packed cell volume, tumor volume, inhibiting EAC (Ehrlich ascites carcinoma), was investigated in vivo and increases the life expectancy of the victim, protecting the liver of the EAC tumor-bearing mice. The antitumor mechanism functioned by the initiation of PP2A by ceramide causing apoptosis, as is indicated by a structure analogous to phytosteroids [118]. The main criteria to prove the value of the anticancer agent is increasing the age of the animals [310].

### 2.12. Methanolic Extract of Bauhinia Racemose Plant

The methanolic extract of Bauhinia racemose plant (MEBR) from the family of Caesalpiniaceae shows a significant inhibiting activity on the HeLa cancer cell line by decreasing the cell viability *p* < 0.001, IC_50_ = 80 μg/mL in reference to tamoxifen, which accounts *p* < 0.001, IC_50_ = 48 μg/mL according to the concentration dependency. The methanolic extract of Bauhinia racemose plant (MEBR) shows cytotoxic activity against the HeLa cancer cell line and leads to apoptosis. The TPC, or total phenolic content, obtained from the dried extract of the Gallic acid calibration curve is high in value, 886.8252 mg GAE/g. When the concentration of extract becomes high, membrane permeability will decrease and with separation of cells from the well surface will become either necrotic or circular and have a condensed cytoplasm of the clumped cells, a significant characteristic of showing a concentration-dependent anti-HeLa cancer cell line cytotoxic effect of the extract. To determine the degree of comparative cytotoxic potential, the half maximum inhibitory concentration (IC_50_) values of both tamoxifen and MEBR must be known. An image from an inverted fluorescent microscope shows cancer cells liable to MEBR and stained with Dichloro-dihydro-fluorescein diacetate in diacetate; it is unstructured morphology and releases bright fluorescence because of the reactive oxygen species production and intracellular mass formation [311].

## 3. Conclusions and Future Directions

Increasing awareness of phytochemicals and the use of phytochemicals for medicinal activity has led to the development of phyto medicine research. In this review, we provided an account of the phytochemicals utilized for the treatment of cancer using in vitro and in vivo studies. Plants are used to produce medicine since various synthetic drugs show side effects on the patient, and plant sources are easily accessible and cost-effective. Several active components of medicinal plant have been successfully isolated, screened, and found to be effective in inhibiting or preventing various diseases and cancer. Various studies have been done on plants’ phytochemicals and their activity on various diseases using in vitro and in vivo study methods. Since medicinal plant phytochemicals are utilized for the treatment of cancer, it is essential to further screen and investigate new plants for their phytochemicals, which are very strong and effective in the treatment of cancer.

Medicinal plants are the only natural resource for developing effective, safe, and quality anticancer drugs, although some herbal remedies may cause serious health problems and may have side effects if used without the consultation of a professional or medical practitioner. Sometimes the use of these medicinal plants may interact with regular or other drugs and lead to side effects, for instance, allergies. This review shows the therapeutic potential of thirty-three traditional medicinal herbs/plants. These medicinal plants are highlighted for their potential for anticancer activity. Different phytocompounds extracted from different plants from the Northern Himalaya region are used to treat different cancers and this leads to the aim to carry out a biological and chemical investigation of different plants originating from this region and utilized for anticancer activity. The study of active phytochemicals for the treatment of cancer will help to develop drugs for the safe treatment and cure of cancer in the future health care system. The anticancer medicinal plants that constitute phytocompounds to treat specific cancers can also be investigated for activities in other cancer cell lines and this could be decisive in present and future studies.

## Figures and Tables

**Table 1:** List of medicinal plants, their local name, family, habitat, plant part used, and type of anticancer activity.

Name of Medicinal Plants	Local Name	Family Name	Parts Used	Type of Anticancer Activity	Habitat	Reference
*Semecarpus anacardium*	Marking nut tree, phobi nut tree, and varnish tree, Ballataka	Anacardiaceae	Mature fruit	Anticancer	Sub-Himalayan regions	[89]
*Annona squamosa*	Seetapalam	Annonaceae	Aerial part	Colon and breast cancer	Lower regions of the Himalayas	[90]
*Centella asiatica*	Pegaga	Apiaceae	Leaves	Lung cancer	India	[91]
*Asclepiascura ssavica*	Kakatundi	Apocynaceae	Plant	Anti-nasopharynx human carcinoma	India	[92]
*Catharanthus roseus*	Nayantara, Sada Sawagan	Apocynaceae	Root	Breast cancer	India	[93]
*Calotropis gigantea*	*Dryand*, Erukku	Asclepiadaceae	flowers (flower petals)	Anti-Ehrlich ascites carcinoma cells	India	[94]
*Bidens pilosa*	Kateeli	Asteraceae	Leaves	Oral, liver, colon, and breast cancer	Garhwal, Uttaranchal, India	[37]
*Blumealance olaria (Roxb.)*	Buarze	Asteraceae	Leaves	Anticancer	Uttara Kannada district, Karnataka, India	[95]
*Inula racemosa*	Pushkarmula	Compositae	Roots	Colon, prostate, CNS, ovary, leukemia, and lung cancer	Jammu and Kashmir (India)	[61]
*Mikania micrantha Kunth*, Maotamgui	Maotamgui	Asteraceae	Leaves	Anti-esophageal adenocarcinoma	India	[96]
*Cyanthillium cinereum*	Little ironweed	Asteraceae	Plant	Anti-matrix metallopeptidases	India	[97]
*Vernonia cinerea*	Mookkuthi Poondu	Asteraceae	Aerial part	Anti-oral squamous cell carcinoma and lung carcinoma	India	[98]
*Xanthium strumarium*	Banokra, lanetsuru	Asteraceae	aerial parts (stems and leaves)	Anti-proliferative against HepG2 cancer cells	India, Kashmir	[99]
*Begonia malabarica*	Rathasurai, Kakidziihe	Begoniaceae	Plant	Colon, lung, and stomach cancer	India	[100]
*Arnebia euchroma*	*Johnst*, Ratan jot.	Boraginaceae	Roots/leaves	Anti-tumor	India	[33]
*Croton tiglium*	Koni-bih	Euphorbiaceae	Seeds	Anti-tumor S-180 and Ehrlich	India	[101]
*Euphorbia hirta*	Jar dudli	Euphorbiaceae	Whole plant	Anti-myeloid leukemia cancer cell line	Kashmir (India)	[102]
*Mallotus philippensis*	Rohini	Euphorbiaceae	Fruit Root, hairs	Anti-promyelocytic leukemia cells	India	[103]
*Albizia lebbeck*	Siris tree	Fabaceae	Bark	Breast cancer	India	[104]
*Butea monosperma*	*Taub*., Pala	Fabaceae	Flowers	Breast cancer	Kashmir (India)	[105]
*Vitex negundo*	Nira gundi	Verbenaceae	Leaves	Anti-Dalton’s ascitic lymphoma	India	[106]
*Woodfordia fruticosa*	Dhai	Lythraceae	Whole plant/flowers	Inhibit the proliferation of CML K562 cells	Kashmir (India)	[107]
*Azadirachta indica*	Dhrek	Meliaceae	aerial parts	Anti-MCF cancer Cell	Kashmir (India)	[108]
*Moringa oleifera*	Sajina	Moringaceae	Leaves	Lung cancer	India	[109]
*Syzygium cumini*	Jamun	Myrtaceae	Fruits	Anticancer cell lines ovarian adenocarcinoma, prostate carcinoma, non-small cell lung carcinoma	India, m Keshav Shristi, Bhayandar	[110]
*Phyllanthus emblica*	Sohlhu, aomla	Phyllanthaceae	Fruits	Prevented N-nitrosodiethylamine induced hepatocellular carcinoma	India	[111]
*Plantago major*	Jangremriza, Akaba	Plantaginaceae	Leaves	Against Ehrlich ascites carcinoma	India	[112]
*Potentilla fulgens*	Bajradanti	Rosaceae	Root	Breast cancer	India	[33]
*Citrus limon*	Kanji nemu	Rutaceae	Fresh fruits	Colon cancer	India	[113]
*Taxus wallichiana*	Lauthsalla, barmi, banya	Taxaceae	Needles, bark, root, seed, heartwood	Anti-liver, colon, ovarian, and breast cancer cell	India, Kashmir (India)	[114]
*Curcuma longa*	Haldi	Zingiberaceae	Rhizomes	Dalton’s lymphoma cells	India, U.P. Himalaya	[115]
*Leguminase*	Siris, Shiris in Hindi; Lebbeck tree in English	Albizzia lebbeck (Lin.) Benth	Bark	Anti-human breast cancer cells	India	[104]
*Anona squamosa*	Gandagatra	Annonaceae V. N. Sitaphala	Plant	The inhibited proliferation of HL-60 cells	India, U.P. Himalaya	[116]
*Asparagus racemosus*	*Wild* Shatavari, Ekalkanto	Liliaceae V. N. Satavari	Roots	Anti-breast cancer, colon, adeno carcinoma, kidney carcinoma, and EAC tumor cells	India, U.P. Himalaya	[117]
*Bacopa monniera*	Water hyssop, water hyssop, brahmi	Scrophulariaceae V. N. Brahmi	Plant	Ehrlich ascites carcinoma	India, U.P. Himalaya	[118]
*Bauhinia racemosa*	Asundro Bidi Leaf tree	Caesalpiniaceae V. N. Kandi	Bark	Against a HeLa cancer cell	India, U.P. Himalaya	[119]
*Berberis asisatica*	Daruharidra, Darbi (Sans)	Berberidaceae V. N. Kingori	Roots	Dalton’s lymphoma ascites tumor cells	India, U.P. Himalaya	[120]
*Berberis aristate*,	Daruharidra	Berberidaceae V. N. Daruharidra	Bark	Anticancer	India, U.P. Himalaya	[121]
*Berberis lyceum*	Ishkeen (Kashmal and Darbald)	V. N. Kingore	Root	Anticancer	India, U.P. Himalaya	[122]
*Bergenia ligulata*	Pakhanbheda	Saxifragaceae V. N. Silparo	Roots	SARCOMA-WM1256 IM	India, U.P. Himalaya	[123]
*Calotropis gigantea*	Milkweed, Madara, Akavan, Aak	Asclepiadaceae V. N. Akha, Arka	Root, leaves & Latex	Prevents oral, prostrate, and colon cancer, anti-hepatocellular carcinoma	India, U.P. Himalaya, India, U.P.	[124,125]
*Cedrus deodara*	Dabadru, Devadru, Devadrus, Devdar (Bengali)	Pinaceae V. N. Devadara	Heartwood	Anticancer	India, U.P. Himalaya	[126]
*Datura metel*	Indian Thornapple, Hindu Datura	Solanaceae V. N. Dhatura	Aerial parts	Anti-colorectal carcinoma	India, U.P. Himalaya	[127]
*Podophyllus emodi*	Himalayan/Indian may apple	Berberidaceae V. N. Baryakarkatee	Rhizomes	Anticancer	India, U.P. Himalaya	[128]
*Urtica dioica*	Nettle leaf, or just a nettle or stinger	Urticaceae V. N. Bichubooti, Brishakali, Kandali	leaves and stems	Repress prostate-cell metabolism and proliferation	India, U.P. Himalaya	[129]

**Table 2:** Chemical structure and the specific activity of major anticancer phytochemicals extracted from different plants.

Species Name	Phytochemicals	Chemical Structure	Types of Cancers	Reference
*Catharanthus roseus*	Vinca alkaloids	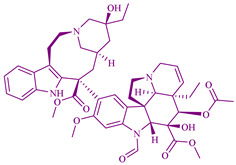	Kaposi’s sarcoma, breast, leukemia, testicular and lung cancers, and lymphomas	[130]
*Podophyllum peltatum*	Podophyllotoxin	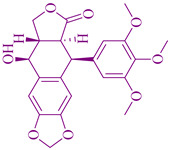	Testicular, bronchial, and lymphomas cancer	[131]
*Taxus brevifolia*	Taxanes	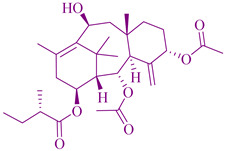	Kaposi sarcoma, lung cancer, ovarian and breast cancer	[132]
*Camptotheca acuminata*	Camptothecin	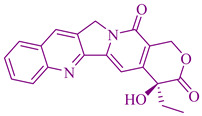	Colorectal, lung, ovarian cancer	[133]
*Cephalotaxus harringtonia* var. *drupacea*	Homoharringtonine	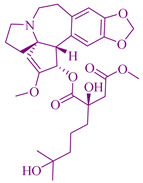	Chronic myelogenous leukemia and myelogenous leukemia	[134]
*Albizia lebbeck*	Saponin	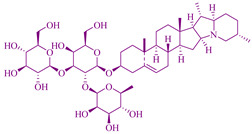	Cervical, larynx, hepatocarcinoma, breast, and colon carcinoma	[135]
*Annona squamosa*	Isoquinoline alkaloid	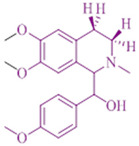	Liver, breast, and colon cancer	[90]
*Arnebia euchroma*	Shikonin	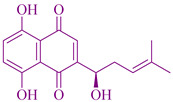	Prevents cancer-causing and malfunction compounds	[33]
*Asclepias curassavica*	Calotropin	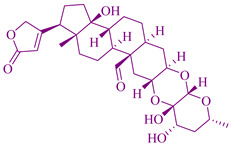	Nasopharynx carcinoma	[136]
*Asparagus racemosus*	Shatavarin IV	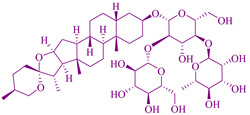	Ehrlich ascites carcinoma (EAC) tumor	[137]
*Bacopa monnieri*	Stigmasterol	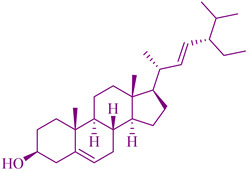	Inhibits EAC (Ehrlich ascites carcinoma) and protects the liver of the EAC patient	[118]
